# Participatory research for the development of information, education and communication tools to promote intermittent preventive treatment of malaria in pregnancy in the Democratic Republic of the Congo, Nigeria and Mozambique

**DOI:** 10.1186/s12936-021-03765-4

**Published:** 2021-05-19

**Authors:** Sylvain Landry Birane Faye, Maud Majeres Lugand

**Affiliations:** 1 Laboratoire de Sociologie, Anthropologie, Psychologie (LASAP), Department of Sociology, Cheikh Anta DIOP University (UCAD), Dakar, Senegal; 2 Social Research Manager, Access and Product Management, Medicines for Malaria Venture, Geneva, Switzerland

**Keywords:** Sub-Saharan Africa, IPTp, Pregnancy, Malaria, Communication tools, Sulfadoxine–pyrimethamine

## Abstract

**Background:**

To improve the coverage of intermittent preventive treatment of malaria in pregnancy (IPTp) in Africa, Medicines for Malaria Venture (MMV) developed, tested and validated a new packaging of sulfadoxine–pyrimethamine (SP), as well as specific communications tools designed to improve knowledge of IPTp and the motivation of women to adhere to it, particularly if it is distributed by community health workers (CHW).

**Methods:**

This article describes and analyses the results of an empirical research carried out in the Democratic Republic of the Congo (DRC), Nigeria and Mozambique, to evaluate the perception and social acceptability of SP for healthcare providers, CHW and pregnant women, and to assess the ability of the new SP packaging and the communications tools to change their perception of SP and improve their attitudes towards IPTp.

**Results:**

The results indicate that SP’s new individual packaging was perceived by pregnant women and healthcare providers as a “hygienic” and “safe”, with a specific identity. The graphics used in IPTp communications tools were modified according to the respondents’ feedback to make them more culturally and socially sensitive, and then validated. However, although the new blister packaging and IPTp communications tools generated greater confidence and motivation, SP side effects as well as preconceived ideas, particularly regarding its efficacy, remain a challenge that must be addressed to improve IPTp acceptance and compliance by healthcare providers and pregnant women.

**Conclusion:**

This participatory approach to social research based on ongoing feedback to the graphic designer provided more empirical evidence to improve and adapt the textual and visual content of communication tools (SP blister packaging, leaflet, user guide) to local contexts and user preferences. Tested and validated in different socio-cultural and socio-political contexts, these tools provide a good basis for the promotion of IPTp in Africa.

## Background

Although the scaling up of malaria interventions has helped reduce malaria deaths worldwide, this disease remains a concern in Africa. In 2019, with an estimated 229 million malaria cases and 409,000 malaria deaths, the World Health Organization (WHO) African Region accounted for about 94% of cases and deaths globally [1]. In the same year, 11.6 million pregnancies out of 33.2 million (35%) were exposed to malaria infection in sub-Saharan Africa [1], the highest prevalence being in Central Africa (40%), closely followed by West Africa (39%), and East and Southern Africa at 24% [2]. In pregnant women, malaria can lead to anaemia, maternal death, preterm delivery low birth weight, and stillbirths.

Since 1998, the WHO recommends the administration of sulfadoxine-pyrimethamine (SP) for IPTp in all eligible pregnant women during antenatal care (ANC) visits, starting as early as possible in the second trimester of the pregnancy [1]. Each dose of SP should be administered at least 1 month apart, and each woman should receive at least three doses (IPTp3) during her pregnancy for optimal protection [3]. In 2012, SP combined with amodiaquine was also introduced to prevent malaria in children in the Sahel regions where transmission is seasonal [4]. Self-medication with SP is still commonly used for uncomplicated malaria and it is still widely supplied in the private sector. Due to its extensive use, parasites have become increasingly resistant to the drug in the context of treatment and the social acceptability of SP for IPTp has become problematic [5].

A recent systematic review and meta-analysis [6] suggest a decline in the effectiveness of IPTp for reducing malaria infection with increasing resistance. Nevertheless, the use of SP during pregnancy remains associated with reduced risks of low birth weight, anaemia [7–10].

While waiting for future clinical trials to test the efficacy and safety of other antimalarial drugs to prevent malaria during pregnancy, the WHO still recommends continuing using of SP for IPTp, especially in moderate to high transmission areas. However, IPTp coverage, which means that the woman consumes at least three doses of SP during pregnancy, from the second quarter in particular is still low in sub-Saharan Africa. In 2019, in 33 countries which have adopted IPTp in their policy, 80% of all pregnant women visited ANC clinics at least once during their pregnancy, of which 62% received at least one dose of IPTp, 49% received at least two doses of IPTp, and only 34% received the minimum recommended three doses of IPTp [1]. Contrary to popular belief, low IPTp coverage is not solely attributable to low ANC attendance [11] or the socio-economic characteristics of women [12]. Other factors include that ANC clinics do not run optimally due to limited training, low motivation of some healthcare providers and regular stock-outs [13], and remote access for pregnant women [14, 15]. In Malawi, Ghana, and Kenya, negative interactions with healthcare providers during ANC visits, experiences of side effects, and negative perceptions of SP by pregnant women also influence its use [16]. Exacerbating this, the "off-label" use of SP may have created doubts about the quality of the product, leading to acceptability issues [14, 17]. Some authors believe that simplified educational messages would improve the acceptability of the drug by pregnant women [18]. Previously SP was only available in bulk in 1000 tablet jars, so developing individual packaging, specifically supporting the IPTp indication, could help improve the perception, acceptability, and use of SP [19, 20].

Medicines for Malaria Venture (MMV) received a grant from Unitaid to support the development of new, high-quality SP formulations, prequalified by the WHO. It developed graphic information for the primary (blister) and secondary (dispensing box) packaging for pregnant women to be distributed by community health workers (CHW). In addition, a user guide for community relays and a leaflet for pregnant women were designed with simplified graphics and pictograms communicating on IPTp, SP, and ANC. This project focusing on the supply side of IPTp was in complement with the "Transforming IPT for an optimal pregnancy" (TIPTOP), a Unitaid funded project, managed by Johns Hopkins Program for International Education in Gynecology and Obstetrics (JHPIEGO), assessing the impact of the integration of CHWs on IPTp coverage in the DRC, Madagascar, Mozambique and Nigeria.

The assumption made here is that an individual SP packaging with a specific IPTp indication, no longer recommended for the treatment of uncomplicated malaria, would help correct misinformation, generate a more positive image, and could improve IPTp acceptance and compliance by the drug providers with limited training and by women with little access to information on IPTp. Assuming the community level context where the IPTp intervention will be made available, a participatory research approach [21] involving end-users from the communities was chosen. This iterative qualitative method allowed them to directly contribute by sharing their views and opinion on IPTp intervention, SP and its packaging presentation, as well as related communication tools. The outcomes of the discussion were used to inform the development of user-friendly and appropriate communication tools. This approach is also viewed as an essential component of ethical good practice in research and brings community health benefits through ensuring the success of an intervention [22].

Participatory research is a collaborative, iterative design that focuses on a “process of sequential reflection, planning carried out with and by local people rather than on them and emphasizes direct engagement of local priorities and perspectives [21, 23]. This approach allows co-constructing research through partnerships between researchers and stakeholders, community members, or others with insider knowledge and lived expertise. It uses systematic inquiry in direct collaboration, which allows research to benefit from the collective wisdom of both. In turn, it creates more meaningful findings translated to action or for the purpose of social change [24].

The key difference between participatory and non-participatory techniques lies in community dialogue, partnership, and handing power from the researcher to research participants [21, 25]. It is known as an essential component of ethical good practice in research and health programmes and brings community health benefits through ensuring the success of an intervention [22, 26]. Partnership with communities is critical for developing information, education, and communication (IEC) tools to promote health-related products.

Nevertheless, some researchers raise the various challenges that make a fully participatory project unrealistic [21, 22, 26–28]. Especially, the issue of the reality of power imbalance between researchers and the community under study, can limit their capacity to give input in ways that extend beyond responding to researcher plans or agendas [25, 27, 28].

The field research using participatory approach was conducted in the DRC, Nigeria, and Mozambique, countries where community IPTp pilots were on-going, to address the following questions:

- How did the healthcare providers, CHWs, and pregnant women view the SP packaging (format, visual aids, the information conveyed)?
- Were the key messages and pictures contained in the communication tools clearly understood and answered their preoccupations?
- Did it influence their perceptions and attitudes towards SP and IPTp?
- Were they acceptable to the population and likely to enhance their IPTp compliance?
- And if not, how can its acceptability be improved?

The attitudes, points of view, and knowledge of the participants regarding the SP packaging and the IEC tools were documented, and the potential influence of these tools to improve the users’ perceptions of SP and their IPTp compliance was analysed. The added value of the collaborative approach between the graphic designer (tools designer) and the social research teams (as the relay) to test tools with end-users and adapt them to local contexts and preferences is also discussed.

## Methods

### Study design

The qualitative study combined in-depth individual interviews (IDI), focus groups discussion (FGD), and direct observations, which offers the advantage of cross-referencing what participants say about SP with their actual practices. Furthermore, the points of view put across in the individual interviews were compared with the data provided by the FGD to interpret the information. IEC tools thus were developed using an iterative and collaborative process. They were first produced by a graphic designer, with the help of the TIPTOP consortium, before being tested empirically. The discussions tested the understanding of the tools by pregnant women and community relays, without giving them any explanation beforehand, to identify the gaps and make the appropriate changes. Results of these tests were analysed with local teams and participants daily, shared with the graphic designer (remotely based in Geneva) on an ongoing basis, who took the insights collected into account to produce updated versions to be retested, and validated by participants. The researchers interacted directly with community participants on the field, by listening to them, and by collecting their opinions on how tools could be improved.

### Study sites

The research was conducted in the DRC, Nigeria, Mozambique, three countries hosting the IPTp TIPTOP project and characterized by low IPTp coverage and high rates of malaria infection among pregnant women. For example, in the DRC, 69% of pregnant women received IPT2 and 34.5% IPT3 [29]. The research was conducted first in the DRC (February 2018), then in Nigeria (June 2018), and finally in Mozambique (July 2018).

### Recruitment and data collection

In each country, a district was selected in an area with low IPTp3 coverage and with an IPTp3 coverage rate lower than IPTp1 (IPTp 1, 2, 3 correspond to the respective first, second and third recommended dose). The sampling used either routine or national data available in each country, or the results of baseline surveys carried out by JHPIEGO in each of these countries as a prelude to the implementation of the TIPTOP project. Kenge (Kwango, DRC), Nhamatanda (Sofala, Mozambique), and Ohaukwu (Ebonyi, Nigeria) were selected. In each district, three health zones were chosen for their higher number of pregnant women, identified by the local health authorities in ANC clinic registers. In each health zone, a locality was selected based on the same criteria, and households were identified with the help of the CHWs. Healthcare providers were selected to be interviewed based on their involvement in maternal and child health programmes.

At the household level, the research targeted primarily pregnant women living in the selected localities, their husbands, as well as women who had recently given birth less than 6 months before. CHWs and health authorities were part of the sample. A total of 294 people were interviewed in all three countries, with 103 individual interviews and 191 through focus groups.

### Data analysis

Data were collected in each country by a local team of investigators recruited remotely, proficient in the local languages (students in sociology or community health workers), trained by senior researchers (health public specialist, anthropologist). The individual interviews and focus groups were transcribed from local languages into French (DRC) and English (Nigeria, Mozambique). The data was exported to the ATLAS Ti software to be encoded and categorized. Thematic content analysis was used and themes generated from the data based on a mixed approach. First, a deductive coding was carried out based on the major variables of the survey for each country. Then, it was completed by an inductive coding, based on new themes that emerged from the fieldwork and the transcripts. Results were triangulated according to the tools and targets investigated: the coding process was applied for data individual interviews and focus group. Also, the data interpretation took into account the variability of interviewed categories. By cross-referencing their views concerning a theme, the interpretation of the data should be enriched.

A report was generated per country after completion of field work in respective countries. This was complemented by a synthetic analysis of the three data sets recognizing the various disparities between them including, health systems in place, infrastructural, social and cultural contexts limiting the conclusion. The attempt of this piece of research was to generate evidence from various end-users’ perspectives on IPTp intervention and to identify ‘universal’ pictograms and visual aids contextualized, which could be understood by the majority of the respondents.

### Ethical approvals

To conduct the surveys, the approval of the ethics national committees for research was obtained, at the national (Kinshasa), state (Nigeria, Ebonyi) or university (Maputo) levels. During data collection, an information sheet and the consent form were read and shared with each respondent for approval.

## Results

The analysis highlighted major themes across the data collected: (a) the perceptions of IPT for malaria prevention by pregnant women and CHWs, especially attitudes towards SP drugs; (b) the opinions on the SP blister for IPT in pregnant women; (c) the comprehension, opinions, and acceptability of the of communication materials and (d) the willingness and ability to change SP use behaviour.

### Perceptions, attitudes towards IPTp, SP drugs influence communities adherence to recommended doses

In all three countries, very few women had taken the required 3 doses of SP during pregnancy. The identified contributing factors included the negative perceptions of SP and side effects which did not motivate women to take all three doses.

*I only had the first dose and I threw away the next one. I did not take it anymore during my pregnancy because it made me dizzy. Also, some people said that the tablets were not hygienic, and others said that it could affect the health of my child.* FGD_DRC_Kenge_Barrière_Sas_Woman_E3

Also, some women expressed doubts about the hygiene and safety of SP. White tablets without any distinctive sign make them undistinguishable from counterfeit drugs, which are suspected of harming the pregnancy’s outcome. Moreover, the unreliability of the ANC clinics due to SP stock-outs (DRC, Mozambique) creates missed opportunities for IPTp.

*A woman can turn up to the appointments given to her. But when she gets to the health centre, she notices that there is a shortage of drugs.* IDI2_DRC_Kenge_Health Authorities_Nurse Supervisor

Besides, some women did not take all three SP doses due to a lack of information given by the healthcare providers. The latter expected to promote IPTp often have doubted themselves about SP, and its effectiveness for preventing malaria in pregnancy, and as a result do not talk about it during the consultation with the patient. Also, the importance of socio-cultural practices and traditional medicine making pregnancy a social event hurts ANC attendance and access to SP. Taking drugs to prevent a disease that has not happened is not taken seriously, and should the women become ill, plants and herbs would be preferred, in line with local emic representations of malaria.

### Health workers and pregnant women’s opinions, attitudes related to the new SP blister packaging

**Fig. 1:**
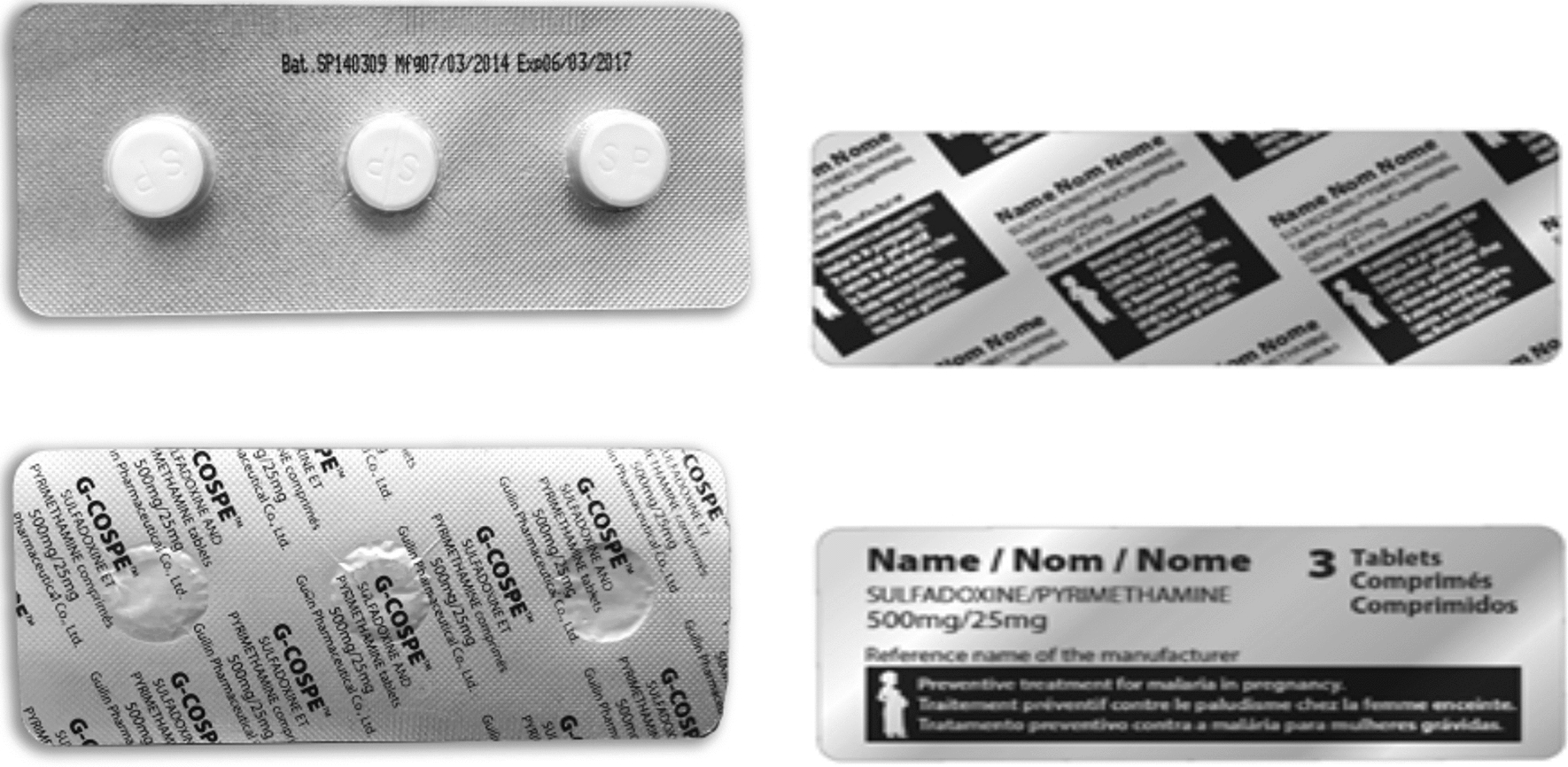
SP blister packaging tested in the DRC and improved for Nigeria and Mozambique

In the Kenge district (DRC), the first version of the SP blister (see Fig. 1) was tested in the Barrière and Saint-Esprit health areas.

The SP blister was seen as safe and practical, offering better protection against rain or dirt, but it also provides information (expiry date) that can reassure women.

*“What interested me the most here was to know the expiry date of the product, because with tablets in bulk, the woman who takes the product does not know it. We give her only the 3 tablets”* IDI_DRC_ Kenge area chief medical officer.

Compared to SP distributed in bulk, the blister packaging makes the tablets more hygienic and ensures a correct dosage. However, the women noted that the blister did not have easily recognizable distinctive signs identifying the tablets as a malaria prevention product for pregnant women. Furthermore, as white SP tablets are undistinguishable from other drugs and paracetamol in particular, they asked for coloured tablets (e.g. pink), and for diagrams representing mosquitoes and a pregnant woman on the packaging.

**Fig. 2:**
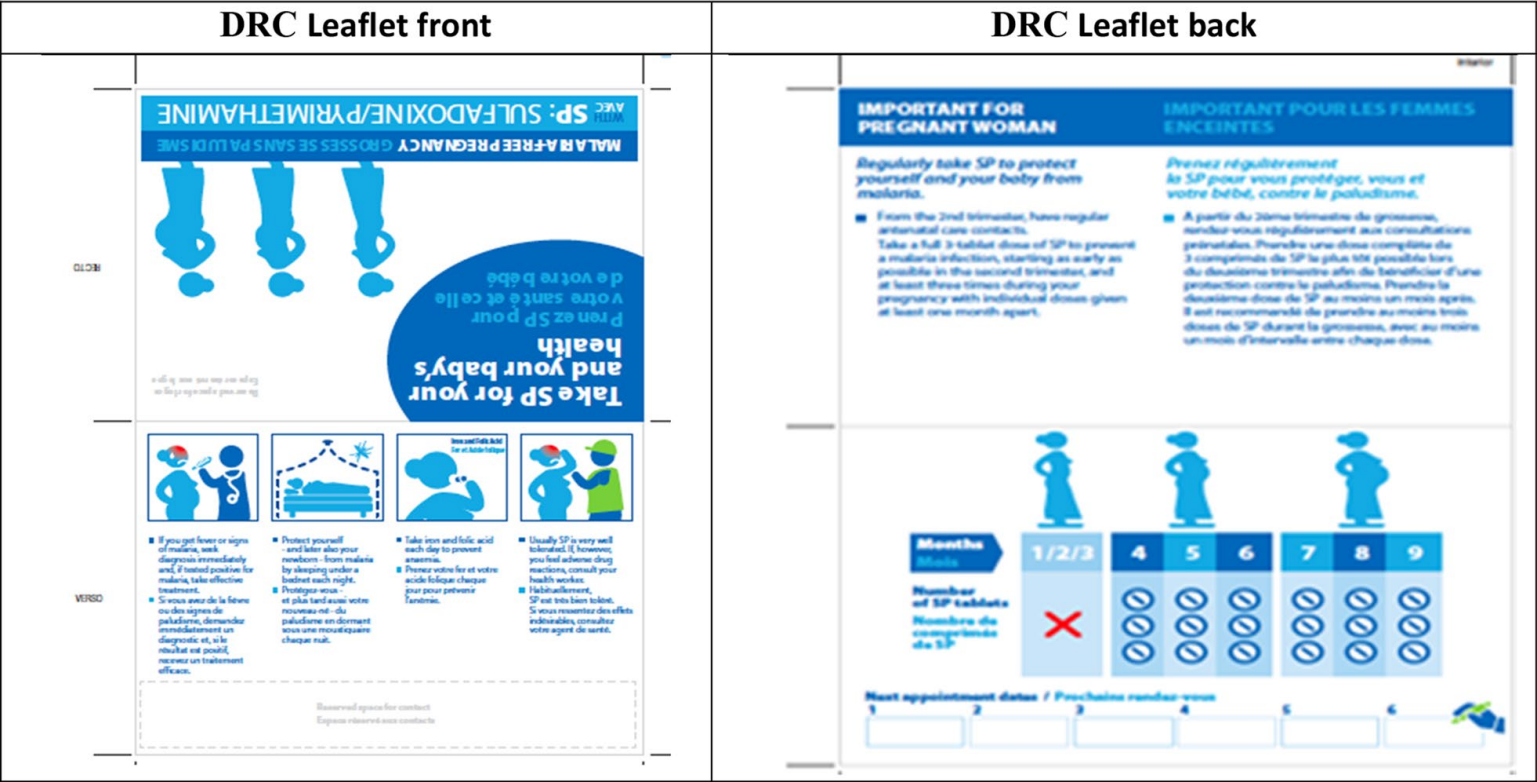
Revised leaflet for pregnant women tested in DRC

**Fig. 3:**
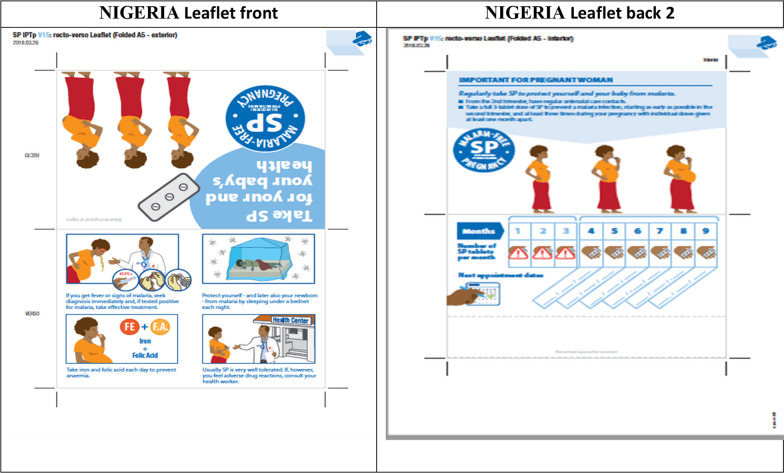
Revised leaflet for pregnant women tested in Nigeria

**Fig. 4:**
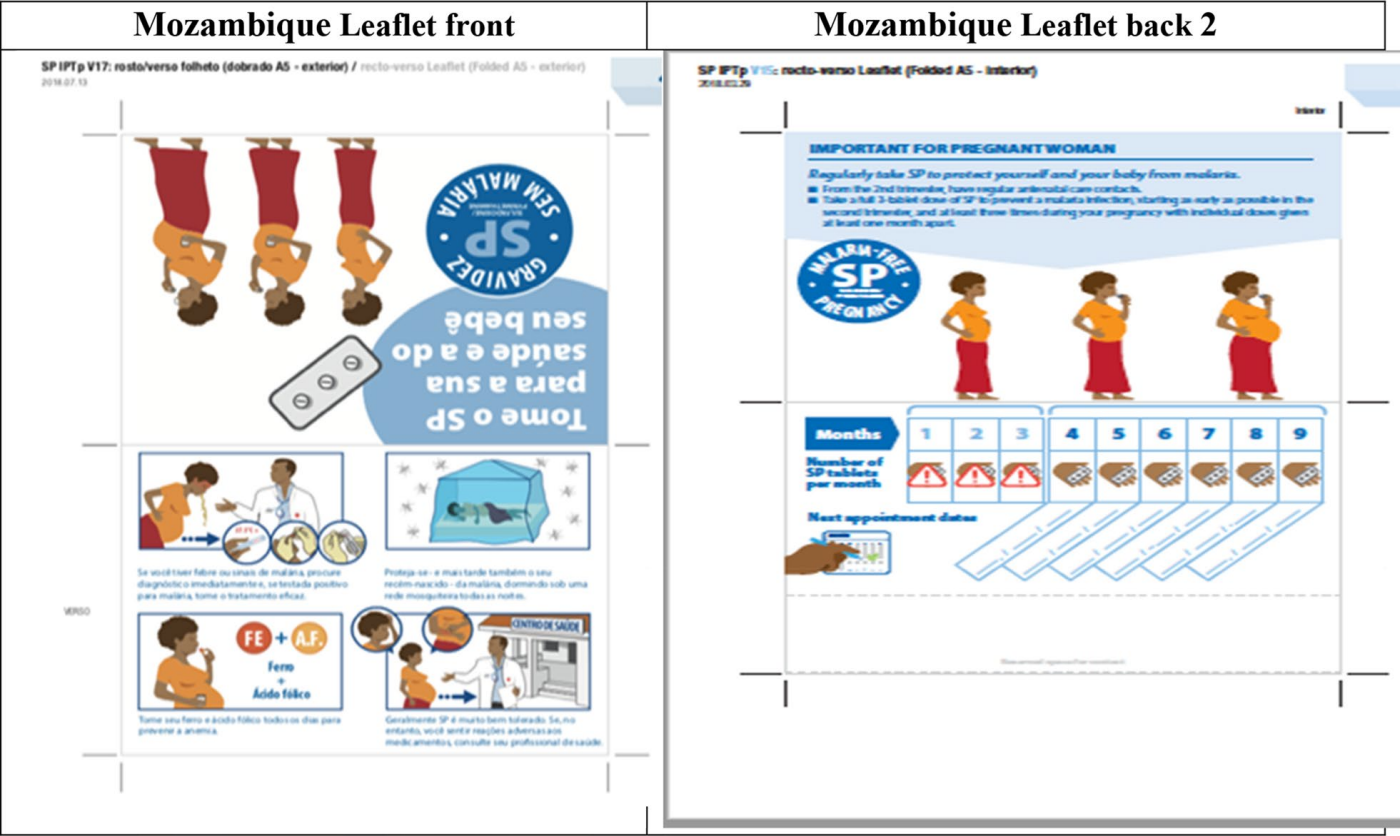
Revised leaflet for pregnant women tested in Mozambique

The graphic designer produced a new version of the blister, taking into account some of these suggestions, which were tested in the DRC (Mukila), then in Nigeria and Mozambique (see Figs. 2, 3, 4). The new packaging was perceived positively by women. In Nigeria, the new blister helped women distinguish this drug from counterfeit drugs suspected of harming the pregnancy’s outcome. The larger picture of the pregnant woman was more easily recognizable and the text on the blister was also useful for those who could read. A suggestion was made to put a logo on the blister pack to reinforce its identity, as well as a short message service (SMS) code or telephone number to check whether it is counterfeit or not.

### The participatory design process of health-related product to promote IPTp

Discussion with community participants helped to understand their opinions, and acceptability of the communication materials. The user guide for CHWs and a leaflet for pregnant women promoting IPTp were tested. As they were based on the same central design, the leaflet will be used to discuss results.

In the DRC (see Fig. 2), the pictures of the people were too abstract to identify them. The picture of the woman with a neck not connected to the rest of the body was criticized. Her position lying on her back under the mosquito net was contrary to local habits (pregnant women lie on their side). Furthermore, the picture did not show accurately the action of taking the medication or of attending the ANC clinic. There was also too much text on the leaflet, as women were mostly illiterate. The health authorities noted that, although the leaflet provided information on SP, iron, folic acid and insecticide-treated mosquito nets, no mention was made of mebendazole, the 4th product recommended in the essential practices for child health in the DRC.

In Nigeria (see Fig. 3), the revised version messages were understood properly by the women, particularly the prohibited use of SP during the first trimester of pregnancy. The front of the leaflet was perceived as clearer and easy to read, with less text and pictures easy to understand. The pictures of iron and folic acid were easily identified by the colour of the tablets, well known to the women. However, the message on the need to consult health services in the event of side effects was not understood spontaneously. CHWs and nursing staff pointed out the lack of messages encouraging regular visits to ANC clinics by pregnant women, even though there was a dedicated section to indicate appointment dates. On the back of the leaflet, the message on the prohibited use of SP during the first trimester of pregnancy (see Fig. 2) was not understood in the first picture. In the second version of the back, the information was understood with a warning sign on the first 3 months and with the pictures of women bringing their hands to their mouths from the fourth month. Also, the link between the appointment and the month was also well understood.

In Mozambique (see Fig. 4), the women understood the messages on the need to go to the health centre in the event of vomiting and fever, and on the prohibited concomitant use of cotrimoxazole and SP (front). On the back of the leaflet, the picture of the pregnant woman taking a tablet with a glass of water in her hand, as of the 4th month, was understood correctly and appreciated, along with the calendar to mark the dates SP is taken during the pregnancy. However, the women suggested that the clothes worn by the woman should be typically African clothing (*capulana*, scarf on the head or dress usually recommended for pregnant women), and that the X sign should be used to reinforce the prohibited use.

**Fig. 5:**
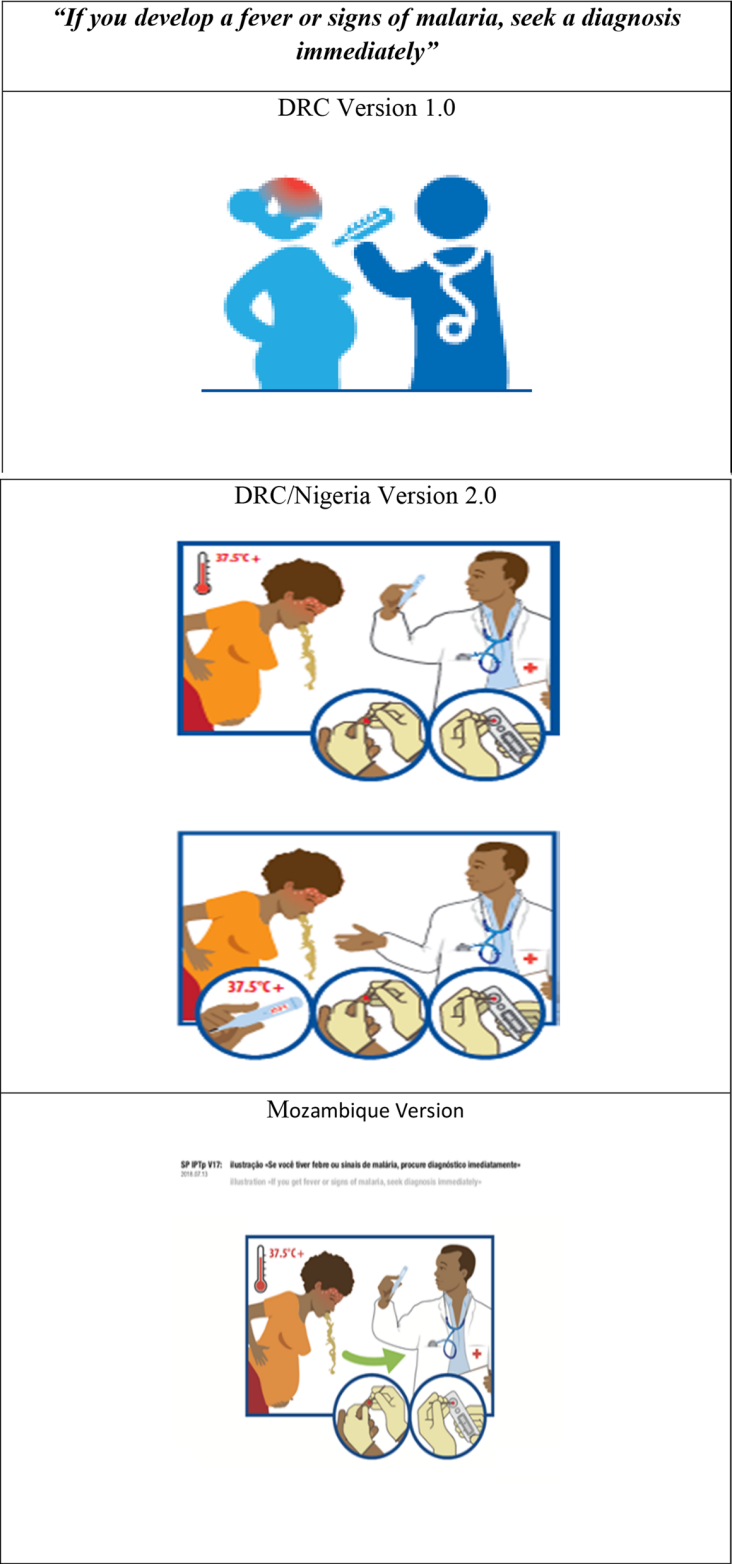
Revised versions of the diagrams in the message “If you develop a fever or signs of malaria, seek a diagnosis immediately” for all three countries

The leaflet for pregnant women included diagrams for key messages. Below, two examples describe the women’s reactions and the changes made to its content as empirical tests were carried out. The first diagram shows the necessity for pregnant women to seek a diagnosis if they develop a fever or signs of malaria (see Fig. 5).

In the version tested in the DRC, the women found it difficult to understand the message. The diagram was generally perceived as a woman with a red forehead and a man with intestinal problems. Respondents did not recognize him clearly as a healthcare provider. The thermometer was thought to be a pregnancy test and the stethoscope was seen as intestines.

**Fig. 6:**
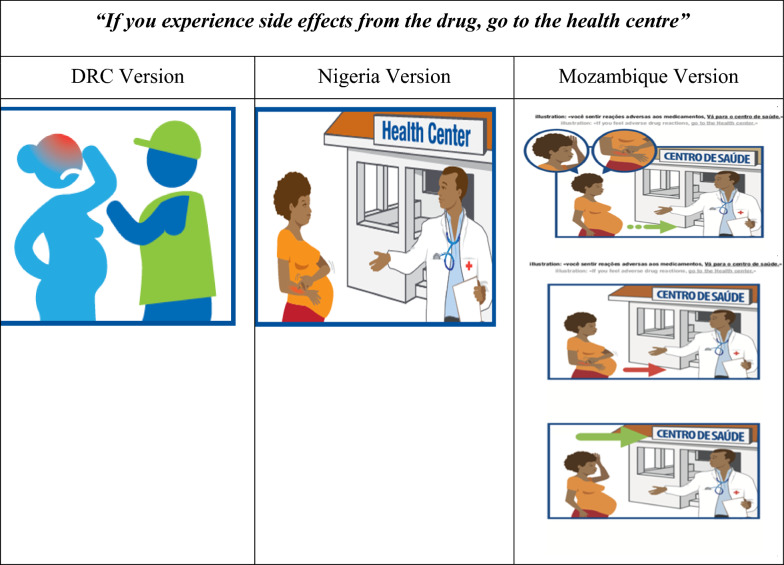
Revised versions of the diagrams in the message “If you experience side effects from the drug, go to the Health centre”

Tests carried out in Nigeria showed that the new version was easier to understand. With this second version, women recognized the signs of vomiting and high temperature (thermometer), the diagnostic test being carried out, and the presence of a healthcare provider. In the Mozambique version, the need to show the diagnostic procedure was taken into account. The user guide included another diagram showing the need to go the health centre in the event of side effects from the medication (see Fig. 6).

In the first version, the picture was thought to represent a CHW, or a road policeman (they wear green vests) talking to a pregnant woman with a fever. Suggestions were made to make the caregiver more realistic wearing a white coat, and to make the visit to a health centre more explicit. In the revised version 2 (Nigeria), the picture of a hand scratching the arm as a side effect was confusing and did not relate to the most common side effects experienced by pregnant women, i.e. headache and vomiting. The picture was also thought to represent a caregiver saying goodbye to the woman after the consultation. In the modified version tested in Mozambique, women recognized several side effects as well as the message to seek medical help at a health centre. The CHWs asked for a backdrop to be added to the picture, to emphasize the health centre.

### The new blister packaging inspires confidence but does not change IPTp behaviour

In all three countries, pregnant women said they were more motivated to take SP, as they trusted its individual packaging to provide a safer and more hygienic product. The difficulties to open the blister pack and problems relating to the tablet’s size, documented in previous studies [30], were not mentioned here. Health providers felt the blister presentation improved their motivation to prescribe SP to pregnant women because it is easier to convince them.

*R: The SP in bulk may be damaged and sometimes the women may not believe you. But a blister packaging is well protected and women can be convinced more easily.* IDI2_DRC_Kenge_Saint-Esprit_Kapanga_CHW

However, although the blister packaging increases women’s trust, it is not enough to bring about a change in behaviour regarding the use of this drug and the way forward. Underlying negative perceptions of SP did not change much, due to women’s experiences of side effects.

*R: I'm glad the tablets are packaged in a blister because it makes things easier. But if you want me to take SP, it's not enough. When I look at the medicine, it's the same, it hasn't changed. If I take it, it will make me vomit like before...* IDI2_Mozambique_Nhamatandha_ Siluvo_pregnant woman.

## Discussion

Several studies [11] have shown that IPTp compliance is low in the three research countries, namely DRC, Mozambique, and Nigeria. Representations around pregnancy and SP, changing SP indication over the years, and the drug’s association with resistance issues and side effects affect the negative perceptions of pregnant women and their IPTp compliance. To improve compliance, the motivation of women and healthcare providers must be improved [29]. With the new SP blister packaging and IPTp communication tools, pregnant women saw this product as hygienic and safe with a specific identity. The impact of communication on the packaging had been demonstrated previously [31]. However, while the blister can motivate women and give them confidence [32], the existence of side effects as well as the widespread SP resistance influence pregnant women’s acceptance. Combining with this situation affecting opinions and health providers’ confidence in recommending its use, it is critical to communicate and sensitize to accompany any intervention optimally.

The pre-tests carried out on the communication tools, in particular on the user guide for CHWs, highlighted the need to use socially and culturally sensitive graphics, with recognizable people and clothes, as well as messages rooted in local health policy guidelines to improve social acceptability and appropriation [33]. While it is necessary to produce generic tools, it is always useful to adapt them to local contexts [34]. National specificities must be taken into account (national policies, community work, clothing and appearance of people represented).

The participatory research utilized to develop and to contextualize health-related information/products in three various countries has been pertinent. Analysing beliefs, social norms; listening to communities understandings, specifics of contexts, were essential to change the design and production of communication tools for IPTp social marketing. It is important to make special efforts in obtaining community inputs in ways that extend beyond responding to researcher plans or agendas.

In the wider global health literature, efforts have been made to assess how participatory approaches have affected the coverage/participation in health interventions. For example, in Laos, using this approach in the Targeted Malaria Elimination programme has contributed to high levels of community participation in mass anti-malarial administration (above 85%) by adapting the approach according to their needs and responses to the various study components [35]. This research didn’t assess the coverage and participation before and after the design of IEC tools, to claim whether promoting them has affected or not their behaviours related to IPTp. This is one of its limitations, which will be overcome as the tools will be used in real-life as part of the final phase of TIPTOP project. Data on end-users experiences with the updated packaging and additional materials will be collected during the end-line household survey of the pilot.

Listening to communities’ opinions, involving them helped in improving the communication tools to promote IPTp. This approach allowed to make the contents context-specific, and update them based on the feedback, the understanding of the messages improved by participants. It helped to ensure that the contents, images, illustrations reflect the perspectives, cultures, priorities, or concerns of those who are targeted. Validated in different socio-cultural and socio-political contexts, the additional tools such are the user-friendly patient information or CHW job aid, should they be adopted, are a basis and could be adapted based on the context (e.g. local languages, programmes in place). Their implementation is expected to be combined with community relays training and information provided to pregnant women during ANC visits, to reinforce their knowledge.

The iterative collaborative approach with a reworking of the tools by the graphic designer, based on the respondents’ feedback from the field, has proved to be effective. Interactions throughout the study provided much more empirical evidence to guide the production of the tools [36]. This study showed how communication material (blister packaging, leaflet, user guide) can evolve when technical teams work hand in hand with researchers [37]. Nevertheless, even community participants have been able to contribute, and feedback incorporated to the extend possible, final packaging design will have to meet regulatory criteria which could restrict usage of some pictorials/wordings. These challenges remind us that a balance needs to be found in the field between ensuring that the core aims of participatory research are maintained [28], while “it is nearly impossible to create a 100% participatory project” [38].

## Conclusion

The qualitative research included testing the communication tools and updating them by anticipating user needs and making them user-friendly and adapted to social contexts. However, while the SP blister packaging and communication tools adapted to local contexts are useful to motivate healthcare providers to distribute the product at community level and pregnant women to adopt IPTp, they alone are unlikely to lead to a change in behaviour. For that, further measures will be needed, including improving the offer and the availability of quality-assured SP in health facilities, addressing the problem of SP side effects, and enhancing the low motivation of healthcare workers to deliver IPTp by providing, for instance, appropriate training, a problem that is not always solved by using CHWs for community-based distribution.

## Data Availability

The datasets used and/or analyzed during the current study are available from the corresponding author on reasonable request.

## Abbreviations

ANC: Antenatal care
CHW: Community health worker
DRC: Democratic Republic of Congo
FGD: Focus Group Discussion
IDI: In-Depth Interview
IEC: Information, education, and communication
IPTp: Intermittent preventive treatment for malaria in pregnancy
JHPIEGO: Johns Hopkins Program for International Education in Gynecology and Obstetrics
MMV: Medicines for Malaria Venture
SMS: Short message service
SP: Sulfadoxine–pyrimethamine
TIPTOP: Transforming Intermittent Preventive Treatment for Optimal Pregnancy
WHO: World Health Organization

## References

[1] WHO (2020). World Malaria Report 2020.

[2] Trape JF, Pison G, Preziosi MP, Enel C, Du Lou AD, Delaunay V (1998). Impact of chloroquine resistance on malaria mortality. C R Acad Sci III..

[3] Bloland PB, Ettling M, Meek S (2000). Combination therapy for malaria in Africa: hype or hope?. Bull World Health Organ..

[4] WHO (2012). Recommandation de politique générale de l’OMS : himioprévention du paludisme saisonnier pour lutter contre le paludisme à Plasmodium falciparum en zone de forte transmission saisonnière dans la sous-région du Sahel en Afrique.

[5] Ter Kuile FO, van Eijk AM, Filler SJ (2007). Effect of sulfadoxine-pyrimethamine resistance on the efficacy of intermittent preventive therapy for malaria control during pregnancy: a systematic review. JAMA..

[6] Van Eijk AM, Larsen DA, Kayentao K, Koshy G, Slaughter D, Roper C (2019). Effect of *Plasmodium falciparum* sulfadoxine-pyrimethamine resistance on the effectiveness of intermittent preventive therapy for malaria in pregnancy in Africa: a systematic review and meta-analysis. Lancet Infect Dis..

[7] World Health Organization (2008). Technical Expert Group meeting on intermittent preventive treatment in pregnancy (IPTp) WHO headquarters, Geneva, 11–13 July 2007.

[8] Rogerson SJ, Chaluluka E, Kanjala M, Mkundika P, Mhango C, Molyneux ME (2000). Intermittent sulfadoxine-pyrimethamine in pregnancy: effectiveness against malaria morbidity in Blantyre, Malawi, in 1997–99. Trans R Soc Trop Med Hyg..

[9] Schultz LJ, Steketee RW, Macheso A, Kazembe P, Chitsulo L, Wirima JJ (1994). The efficacy of antimalarial regimens containing sulfadoxine-pyrimethamine and/or chloroquine in preventing peripheral and placental *Plasmodium falciparum* infection among pregnant women in Malawi. Am J Trop Med Hyg..

[10] Shulman CE, Dorman EK, Cutts F, Kawuondo K, Bulmer JN, Peshu N, Marsh K (1999). Intermittent sulphadoxine-pyrimethamine to prevent severe anaemia secondary to malaria in pregnancy: a randomised placebo-controlled trial. Lancet..

[11] Thiam S, Kimotho V, Gatonga P (2013). Why are IPTp coverage targets so elusive in sub-Saharan Africa? A systematic review of health system barriers. Malar J..

[12] Chukwurah JN, Idowu ET, Adeneye AK, Aina OO, Agomo PU, Otubanjo AO (2016). Knowledge, attitude and practice on malaria prevention and sulfadoxine-pyrimethamine utilization among pregnant women in Badagry, Lagos State. Nigeria. MWJ..

[13] Akinleye SO, Falade CO, Ajayi IO (2009). Knowledge and utilization of intermittent preventive treatment for malaria among pregnant women attending antenatal clinics in primary health care centers in rural southwest, Nigeria: a cross-sectional study. BMC Pregnancy Childbirth..

[14] Rassi C, Graham K, Mufubenga P, King R, Meier J, Gudoi SS (2016). Assessing supply-side barriers to uptake of intermittent preventive treatment for malaria in pregnancy: a qualitative study and document and record review in two regions of Uganda. Malar J..

[15] Mubyazi G, Bloch P, Kamugisha M, Kitua A, Ijumba J (2005). Intermittent preventive treatment of malaria during pregnancy: a qualitative study of knowledge, attitudes and practices of district health managers, antenatal care staff and pregnant women in Korogwe District. North-Eastern Tanzania. Malar J..

[16] Mubyazi GM, Bloch P, Byskov J, Magnussen P, Bygbjerg IC, Hansen KS (2012). Supply-related drivers of staff motivation for providing intermittent preventive treatment of malaria during pregnancy in Tanzania: evidence from two rural districts. Malar J..

[17] Pell C, Straus L, Andrew EV, Meñaca A, Pool R (2011). Social and cultural factors affecting uptake of interventions for malaria in pregnancy in Africa: a systematic review of the qualitative research. PLoS One..

[18] Tiendrebéogo J, Koiné Drabo M, Saizonou J, Soglohoun CT, Paraiso NM, Sié A (2015). Factors associated with the poor coverage of Intermittent Preventive Treatment in pregnant women in the Pobè-Adja-Ouèrè-Kétou health zone in Benin (in French). Santé Publique..

[19] Berkowitz M (1987). The influence of shape on product preferences. Adv Consumer Res..

[20] Pantin Sohier G (2009). L'influence du packaging sur les associations fonctionnelles et symboliques de l'image de marque. Recherche et Applications en Marketing..

[21] Cornwall A, Jewkes R (1995). What is participatory research?. Soc Sci Med..

[22] Tindana PO, Singh JA, Tracy CS, Upshur RE, Daar AS, Singer PA (2007). Grand challenges in global health: community engagement in research in developing countries. PLoS Med..

[23] Nitsch M, Waldherr K, Denk E, Griebler U, Marent B, Forster R (2013). Participation by different stakeholders in participatory evaluation of health promotion: a literature review. Eval Program Plann..

[24] Vaughn LM, Jacquez F (2020). Participatory research methods - choice points in the research process. J Particip Res Methods..

[25] Sharp RR, Foster MW (2000). Involving study populations in the review of genetic research. J Law Med Ethics..

[26] Lavery JV (2004). Putting international research ethics guidelines to work for the benefit of developing countries. Yale J Health Policy Law Ethics..

[27] Cargo M, Mercer SL (2008). The value and challenges of participatory research: strengthening its practice. Annu Rev Public Health..

[28] Gray RE, Fitch M, Davis C, Phillips C (2000). Challenges of participatory research: reflections on a study with breast cancer self-help groups. Health Expect..

[29] National Malaria Control Program. Rapport annuel d’activités, Measure Evaluation, USAID, République Démocratique du Congo, 2017.

[30] Faye SL (2012). Improving malaria management through rapid diagnostic tests: appropriation by providers communities (Sénégal)(in French). Bull Soc Pathol Exot..

[31] Underwood RL (2003). The communicative power of product packaging: creating brand identity via lived and mediated experience. J Marketing Theory Practice..

[32] Hill RJ, Fishbein M, Ajzen I (1977). Belief, attitude, intention and behavior: an introduction to theory and research. Contemp Sociology..

[33] Gurviez, P. La confiance comme variable explicative du comportement du consommateur: proposition et validation empirique d'un modèle de la relation à la marque intégrant la confiance,* Actes du Congrès International de l'Association Française de Marketing*, 15, éds Usunier, J.-C., Hetzel, P., Université Louis Pasteur, Strasbourg, 1999; pp. 301–26.

[34] de Sardan JP, Diarra A, Moha M (2017). Travelling models and the challenge of pragmatic contexts and practical norms: the case of maternal health. Health Res Policy Syst..

[35] Adhikari B, Pell C, Phommasone K, Soundala X, Kommarasy P, Pongvongsa T (2017). Elements of effective community engagement: lessons from a targeted malaria elimination study in Lao PDR (Laos). Glob Health Action..

[36] Omona J (2009). Social marketing and the fight against malaria in Africa: population services international (PSI) and insecticide treated nets (ITNS). East Afr J Public Health..

[37] Gallen C, Pantin-Sohier G (2014). Pourquoi et comment innover par le design management ?. Gestion..

[38] Minkler M, Wallerstein N, eds.* Community Based Participatory Research for Health*. San Francisco, Calif: Jossey-Bass Publishers; 2003.

